# Supplementary material for: Development of a computer-aided design software for the quantitative evaluation of aesthetic damage
Source: PLoS One. 2019 Dec 18;14(12):e0226322. doi: 10.1371/journal.pone.0226322 (PMC6919621; doi:10.1371/journal.pone.0226322)
Supplement: S1 Code site — 1. (ZIP) [file pone.0226322.s001.zip › [EESC_jr][Projeto]Relatorio_07_21/reports/templates_zip/gentelella-master/vendors/skycons/index.html]

Skycons


# Skycons

**Skycons** is a set of ten animated weather glyphs,
procedurally generated by JavaScript using the HTML5 canvas tag.


They’re easy to use, and pretty lightweight, so they
shouldn’t rain on your parade:

```
<canvas id="icon1" width="128" height="128"></canvas>
<canvas id="icon2" width="128" height="128"></canvas>

<script>
  var skycons = new Skycons({"color": "pink"});
  // on Android, a nasty hack is needed: {"resizeClear": true}

  // you can add a canvas by it's ID...
  skycons.add("icon1", Skycons.PARTLY_CLOUDY_DAY);

  // ...or by the canvas DOM element itself.
  skycons.add(document.getElementById("icon2"), Skycons.RAIN);

  // if you're using the Forecast API, you can also supply
  // strings: "partly-cloudy-day" or "rain".

  // start animation!
  skycons.play();

  // you can also halt animation with skycons.pause()

  // want to change the icon? no problem:
  skycons.set("icon1", Skycons.PARTLY_CLOUDY_NIGHT);

  // want to remove one altogether? no problem:
  skycons.remove("icon2");
</script>
```

Skycons were designed for Forecast
by those wacky folks at **The Dark Sky Company**, and were
heavily inspired by Adam Whitcroft’s excellent
Climacons. The source
code is available on
Github, and has been
released
into the public domain, so please do with it as you see fit!
♡
